# An App-Based Intervention to Protect Home Care Workers From Heat-Related Health Risks: Protocol for a Randomized Controlled Trial

**DOI:** 10.1177/00469580251405766

**Published:** 2025-12-23

**Authors:** Tiziano Gerosa, Remo Fortunato, Francesca Cellina, Martina Ragettli, Davide Marzorati, Andrea Baldassari, Sara Levati, Azra Karabegovic, Isabel Baumann

**Affiliations:** 1 University of Applied Sciences and Arts of Southern Switzerland, Manno, Switzerland; 2 Zurich University of Applied Sciences, Winterthur, Switzerland; 3 Swiss Tropical and Public Health Institute, Allschwil, Switzerland; 4 University of Basel, Switzerland

**Keywords:** heat waves, health risks, occupational health, home care workers, randomized controlled trial

## Abstract

Climate change is intensifying the frequency and severity of heatwaves, posing growing risks for workers. Home care workers face heightened heat risks due to physically demanding tasks, rising patient needs, frequent travel, and limited access to cooling environments. This study aims to causally evaluate the effectiveness of an app-based intervention (REHEAT strategy) in reducing adverse effects of heatwaves among Swiss home care workers. This multicenter, unblinded, randomized controlled trial (RCT) will involve 350 home care workers from the regions of Northern and Southern Switzerland. Participants will be randomized (1:1) into intervention and control groups, stratified by sex, age group, and geographical location. The intervention group will use the REHEAT app from June to September 2025, while the control group will gain access only after summer 2025. Both groups will be monitored over the summer using a wrist-worn wearable device (smartwatch), online questionnaires, diaries and cognitive tests. The impact of the REHEAT intervention will be evaluated using an Intention-To-Treat (ITT) approach to provide reliable real-world effect estimates, supplemented by Local Average Treatment Effects (LATE) analyses to assess impact based on participant exposure and compliance levels. Primary outcomes include strain accumulation, cognitive performance, emotional distress, sleep, night recovery, and mental and physical health. Outcomes will be analyzed longitudinally with mixed-effects models, while LATE will be estimated using app usage as an instrumental variable in generalized linear mixed models. At the time of submission, recruitment had been successfully completed, with a total of 350 participants enrolled in accordance with the study protocol. The intervention and data collection phases are ongoing, and no outcome data have yet been accessed or analyzed. The findings will inform the development of occupational health interventions in response to heat-related stressors and support climate change adaptation policies for health sector workers.

● Climate change intensifies heatwaves, posing growing risks for exposed workers.

● An RCT protocol assesses an app-based heat adaptation strategy for home care workers.

● A co-designed app provides tailored tips, alerts, and resources for heat protection.

● Tested in a Swiss multi-center RCT with physiological, self-report, and meteo data.

● The strategy and protocol are a scalable to other workforces and contexts.

## Introduction

### Background

During the past decades, human-induced climate change has lead to an increase in the frequency, length and intensity of heatwaves.^1-3^ Heatwaves represent a significant threat to human health,^4-6^ and large body of research shows that children, pregnant women or elderly people are especially at risk of heat exposure.^7-9^

More recent evidence indicates that the working population also faces an elevated risk of heat exposure.^5,10-13^ This is especially true for workers employed outdoors, in environments with high temperatures, or engaged in physically and mentally strenuous tasks.^14,15^ Elevated risk arises both from constraints in the context of work, such as time pressure, and from the intensity of the occupational activity.^16^ Research has shown that adverse effects of heat while working can occur already at temperatures lower than those for which public warnings are issued.^17,18^

Previous research on the effect of heat on workers’ health has mainly focused on the economic sectors of construction and agriculture.^14^ Less is known about female dominated economic sectors such as health care. First studies indicate that among health care professionals, home care workers are particularly at risk.^19^ This is due to their work tasks, which require outdoor commuting between their clients’ residences, working in private—often not air-conditioned—homes, and the performance of physically intense activities, such as lifting people. In addition, they frequently face tight schedules. These working conditions do not only lead to physical strain accumulation but also may affect their cognitive performance, emotional distress, sleep, night recovery, and mental and physical health.^14,20-22^

In addition to these direct effects of heat on health, there may also be indirect effects. Heat waves can lead to an increase in service demand.^23^ In fact, there may be increased requests for additional assistance from elderly, disabled, or chronically ill people, who are particularly vulnerable to heat.^24,25^ Moreover, home care workers often implement preventive strategies to protect their clients from the heat, such as opening windows early in the morning or motivating clients to drink enough water. An increased service demand likely results in an additional strain accumulation among home care workers, who may already experience effects of the heat on their health.^26^

Given the evidence about health risks of heat waves, there is an urgent need to find effective strategies to attenuate these risks among home care workers.^27^ While general heat adaptation strategies are widely known (eg, hydration, restriction of strenuous activities, seeking of shade and cool places), implementing them in the specific context of home care is more challenging.^15^ Previous research indicates, for instance, that sufficient hydration is often not achieved due to time pressure and lack of access to sanitary facilities.^28^

The development and implementation of heat adaptation strategies among home care workers is not only in the interest of workers but also of their employers and society as a whole.^15,17^ Improving the resilience of the health care system during heat waves substantially contributes to the general societal resilience in the context of rapidly advancing climate change.^29,30^

### Home Care in Switzerland

In Switzerland, home care organizations provide professional home care for people of all ages who need support due to illness, disability, aging, or recovery after hospitalization.^31^ Both publicly mandated non-profit organizations and private providers deliver these services.^32^

Home care is individually tailored and includes medical and nursing services such as care assessment, medication management, pain and wound treatment, and monitoring of vital signs. Specialized services cover areas like oncology, palliative, psychiatric, and pediatric care.^31^ Basic care focuses on personal hygiene, mobility, and eating assistance, while household support includes shopping, cooking, cleaning, and laundry. Depending on the region, additional offers such as meal delivery, medical transport, and equipment rental may be available. In 2024, 2971 organizations with around 64 000 employees provided home care services nationwide.

Funding follows a dual model.^32,33^ Compulsory health insurance covers nursing care under standardized tariffs, while patients contribute through deductibles and daily co-payments. The remaining costs are shared by cantons and municipalities, with some expenses potentially passed on to clients. Household assistance is excluded from basic insurance and thus is financed privately or through supplementary insurance.

### Rationale of the REHEAT Study

Despite the recognition of heat-related health risks in the home care sector, home care workers have received limited attention in research on human adaptation to climate change. Existing research programs such as HEATSHIELD (Europe)^34^ or Too Hot To Work (Australia)^35^ mostly focused on male-dominated sectors such as construction, neglecting the unique needs of female workers, who make up the majority of the home care workforce. Consequently, more research is needed to understand, first, how heat waves specifically affect home care workers’ health, and second, to identify heat adaptation strategies that can be effectively implemented in the home care setting.^15,36,37^

The project “Home caRE workers’ Health risks of Exposure to heAT waves. Solutions for human adaptation to climate change in the context of working life extension (REHEAT),” based in Switzerland, seeks to address these gaps. In the REHEAT project, we first assess the effect of heat waves on home care workers’ health, focusing on outcomes such as strain accumulation or mental health. Second, we evaluate the effectiveness of a heat adaptation strategy (REHEAT strategy) developed in collaboration with home care workers and experts from 2 Swiss regions. The REHEAT strategy is grounded in a participatory “living lab approach,” aimed at tailoring recommendations and resources to the lived realities of workers.^38-40^ It will be delivered through a smartphone-based app (REHEAT app), offering guidance on hydration, nutrition, clothing and rest, and providing home care workers with heat protection alerts and local resource maps.

According to the Federal Act on Research involving Human Beings (Human Research Act, HRA),^41^ REHEAT is classified as an “other clinical study,” as it involves providing home care workers with training materials and behavioral incentives to increase their awareness of heat protection and encourage the adoption or consolidation of health-protective behaviors. REHEAT falls under risk category A, as it entails only minimal risks and burdens for participants. Specifically, data are collected solely via surveys and wrist-worn wearable devices (smartwatches), and no invasive or potentially harmful interventions are implemented at any point during the study.

### Objectives

This protocol (Version 1, dated 3 September 2025) outlines a randomized controlled trial (RCT) designed to rigorously assess the REHEAT strategy’s effectiveness in preventing the adverse effects of heatwaves among home care workers in 2 regions of Switzerland. The protocol is developed in accordance with the SPIRIT 2025 statement,^42^ and the corresponding checklist is included in the Supplemental Material.

The primary objectives of the RCT are:

1. To causally assess the effectiveness of the app-based REHEAT strategy in reducing negative heat-related health and well-being impacts among home care workers.
2. To examine whether effects vary by worker characteristics such as age, sex, and geographical location.
3. To evaluate participants’ compliance and satisfaction toward the app-based strategy.

### Hypotheses

In light of the above-mentioned objectives, the following hypotheses are proposed:

1. Overall effect: Among home care workers in Switzerland, the REHEAT strategy will reduce the adverse effects of heat exposure, including: strain accumulation, cognitive impairments, emotional distress, sleep disturbances, limited night recovery, and declines in mental and physical health.
2. Age-related heterogeneity: The REHEAT strategy will be more effective for older workers aged 50 and above, with stronger reductions in heat-related adverse effects expected than for younger workers.
3. Sex-related heterogeneity: The REHEAT strategy will be more effective for female workers, who are expected to experience greater benefits compared to their male counterparts.
4. Geographical consistency: Due to the inclusion of practitioners from both regions in the development of the REHEAT strategy, it will be equally effective across geographical areas, with similar impacts observed in both Southern, Italian-speaking, and North-Eastern, German-speaking Switzerland.
5. User engagement: Due to the participatory living lab approach applied for the development of the REHEAT strategy, participants are expected to show high levels of compliance and satisfaction with the REHEAT strategy.

## Methods

### Trial Design

**Figure 1.**
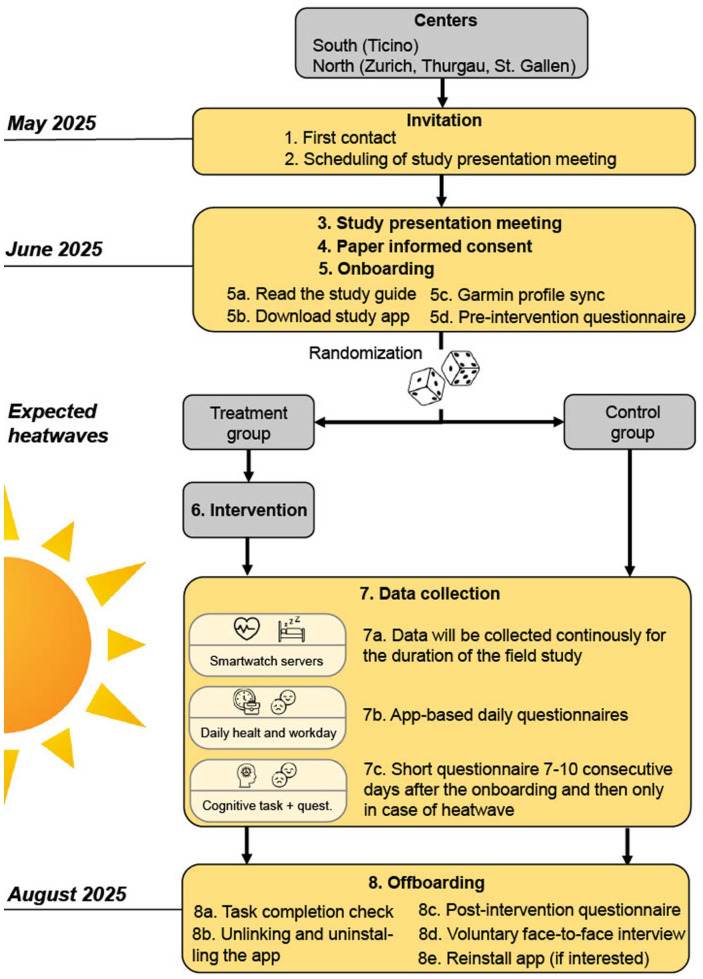
Flow diagram of the REHEAT RCT.

The REHEAT RCT is designed as a 2-arm, parallel-group, multicenter randomized controlled trial (RCT). It involves voluntary home care workers—both employed and freelance—operating in the 2 study centers, 1 in Southern, Italian-speaking, and 1 in North-Eastern, German-speaking Switzerland, including 4 Swiss cantons (Figure 1). The Southern Switzerland study center, coordinated by the University of Applied Sciences and Arts of Southern Switzerland (SUPSI), comprises participants based in canton Ticino. The North-Eastern Switzerland study center is coordinated by the Zurich University of Applied Sciences (ZHAW), which also serves as the sponsor of the trial, and involves participants from the cantons of Zurich, Thurgau, and St. Gallen. The development of the RCT is also supported by the Swiss Tropical and Public Health Institute (Swiss TPH) as a project partner.

The RCT is implemented from June to September 2025, aligning with the period of highest likelihood for heatwave exposure in Switzerland. Participants in the treatment group receive the REHEAT strategy via a smartphone app, while those in the control group use the same app without active intervention features (only tracking of health- and well-being-related parameters) until the end of data collection in September 2025. Although the study is not blinded, all participants install the app during on-boarding activities, to reduce potential bias. To address the risk of contamination—given that participant from the treatment and control group may work together in the same team and share office spaces—participants are explicitly instructed not to discuss or share app content throughout the duration of the study.

This study design enables a robust estimation of the average treatment effect (ATE), as well as its heterogeneity across subgroups. The evaluation is supported by a longitudinal, multi-source, and multi-method data collection framework, which ensures comprehensive measurement of both outcomes and contextual factors over time. The ATE is estimated during the summer of 2025. In the event that no significant heat events occur during the planned study period, a contingency plan allows for an extension of the trial to the summer of 2026 (June-September 2026).

### Participants

Participants are primarily recruited through home care organizations in 2 Swiss regions: the Italian-speaking canton of Ticino in Southern Switzerland, and the German-speaking cantons of Zurich, Thurgau, and St. Gallen in North-Eastern Switzerland. Some of these organizations are public, others private home care providers. Additionally, cantonal institutions and cantonal or local home care associations support the recruitment effort by disseminating the invitation to self-employed workers.

The following inclusion criteria are applied:

- Age 18 or older;
- Employed as a home care worker during the summer of 2025;
- Ability to continuously wear digital wearable device (smartwatch);
- Ownership of a compatible smartphone and willingness to install the REHEAT app.

The following exclusion criterion are applied:

- Having an implanted pacemaker (as this is incompatible with data collection of heart rate variability);

Vulnerable populations (eg, older adults, pregnant workers) are not excluded, as they may benefit most from the intervention. Participation is voluntary, can be terminated by participants at any point without having to provide a reason. All participants are requested to provide written informed consent.

### Recruitment and Consent Procedures

Recruitment for the REHEAT study starts after the approval of the study through the ethical committee of the cantons of Zurich and Ticino on June 6th, 2025. Recruitment takes place through internal communication channels within partner home care organizations and through the presentation of the study among workers of these organizations by the REHEAT research team. Self-employed home care workers are contacted directly using cantonal public lists or through partner associations, while employed home care workers are reached via their employers’ official platforms, such as intranets, internal mailing lists, or presentations in team meetings. A brief announcement introducing the REHEAT study is shared with project representatives within each partner organization and tailored to the communication protocols and preferences of each organization. This message includes a link to the REHEAT webpage, inviting interested individuals to learn more about the study and how to participate.

The REHEAT webpage, available in English, German, and Italian, provides a comprehensive overview of the study, including its objectives, the reasons for focusing on home care workers, the expectations for their involvement, potential benefits, data usage, and detailed instructions for participation. The page clearly outlines the nature and purpose of the study, the procedures involved, its duration, as well as any potential risks, benefits, or discomforts. Clear information is also provided about data protection, including who will have access to personal data and how it will be processed and analyzed.

Contact information for the principal investigators at each study site is available on the REHEAT website, ensuring that all participants can ask questions before deciding to take part. The webpage also includes the study information sheet and privacy policy statement, enabling potential participants to make an informed consent. Participants are asked to download and review both documents, and then confirm—via an online form—their attendance at an in-person study presentation meeting with their designated principal investigator. During this meeting, participants can ask questions, raise any concerns, and decide whether to provide informed consent. If they choose to participate, on-boarding procedures follow. Participants are given at least 1 week to consider their decision before providing informed consent.

The study presentation meetings are conducted in person at the headquarters of each participating organization. During these meetings, the principal investigator and team members present the study, collect signed paper consent forms, distribute sealed, personalized envelopes containing an alphanumeric ID code and a digital wearable device (smartwatch of the company Garmin), and assist participants with the installation of the REHEAT app and the completion of the on-boarding process.

Once signed, paper consent forms are processed in accordance with data protection regulations and securely stored in both physical and digital archives located in Switzerland. Participants may withdraw from the study at any time by informing the research team via the procedure described in the information sheet. Upon withdrawal, the consent is manually deactivated, and any changes are logged in a dedicated electronic register.

As a sign of appreciation, all participants are offered the opportunity to keep the Garmin smartwatch at the end of the study (September 2025). The smartwatch, valuing approximately CHF 120, is provided unconditionally, regardless of compliance with the study’s procedures. The availability of this reward is clearly stated in all recruitment materials. Methodological research on the use of incentives in scientific studies indicates that unconditional incentives are more effective at increasing the participation rate than conditional incentives.^43^

### Randomization and Allocation

Randomization is carried out using a 1:1 allocation ratio through a computer-generated sequence, employing stratified block randomization to ensure balance between key co-variates.

Block randomization within strata works by first dividing participants into homogeneous subgroups (ie, strata) based on key baseline co-variates (eg, age). Within each stratum, participants are then randomized in small, fixed-size blocks to ensure that each treatment group is equally represented in every block. This dual-layered approach helps to maintain overall balance in treatment allocation across important co-variates (through stratification) while also preventing accidental imbalances due to random chance within each stratum (through blocking). It is particularly effective with a limited number of relevant predictors and in samples of small to moderate size, where simple randomization may lead to imbalance.^44^ In our case, we opted for stratification by age (<50 years vs ≥50 years), sex (male vs female), and geographical area (Southern vs North-Eastern Switzerland), and use blocks of 4 to ensure consistent treatment balance within each stratum.

The random allocation sequence is generated by the investigator overseeing the Ticino study site, without involvement from external personnel. Randomization is conducted after the completion of participant recruitment and immediately prior to onboarding and initiation of the intervention phase. Because randomization occurs only after recruitment is finalized, no ongoing allocation sequence requires concealment, eliminating the potential for selection bias due to prior knowledge of group assignments.

Participants in the treatment group receive the REHEAT heat adaptation strategy during Summer 2025 via the REHEAT app, installed on their smartphones. Participants in the control group gain access to the same content only after the summer, in September 2025. As noted previously, group allocation is not blinded. All participants install the study app during on-boarding (June or July 2025); however, only the treatment group has the intervention features activated from the start, while they remain inactive for the control group until the randomized controlled trial ends. Despite limited exposure, there remains some risk of contamination, as participants in both groups work within the same partner organizations and may interact regularly. To reduce this risk, all participants are clearly instructed not to discuss or share the content of the app with colleagues, a condition emphasized as essential to ensure the validity of the study.

### Intervention

The heat adaptation measures of the REHEAT strategy are co-developed by a multidisciplinary team of experts and stakeholders to tailor previously identified heat adaptation solutions to the specific needs and constraints of home care workers and their organizations. The intervention is designed to be low-burden, fully digital, and seamlessly integrated into daily routines.

**Figure 2.**
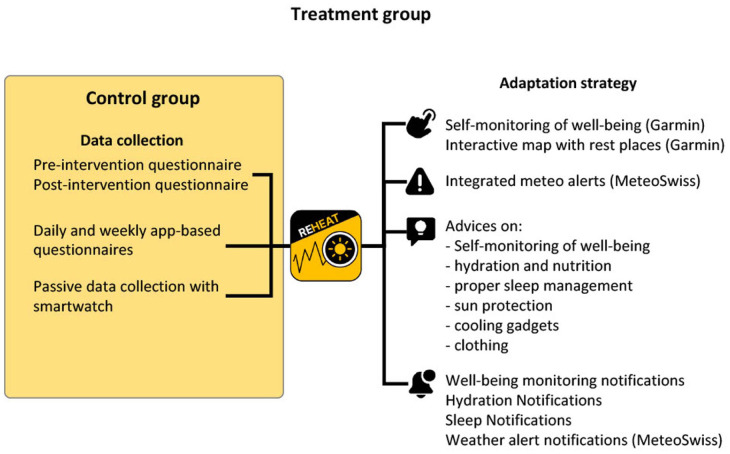
Features of the REHEAT app activated for the treatment group and the control group.

The REHEAT app serves as the delivery platform, bringing the tailored measures directly to participants’ smartphones. For participants in the treatment group, the app provides enriched content from initial on-boarding, including practical guides on self-monitoring, hydration, nutrition, clothing and rest, and providing home care workers with app content reminder notifications (once per week), timely heat protection alerts (from 2 days before the heatwave), and local resource maps (Figure 2). In total, the REHEAT strategy covers 4 intervention domains with 12 heat adaptation features.

1. Personal Care (6 features). This domain focuses on individual well-being and includes a smartphone-based tool for tracking personal well-being, training materials on recognizing and managing heat-related symptoms, and reminders to check physiological data regularly. It offers expert advice on nutrition and hydration during hot weather, complemented with recipes and reminders of hydration, as well as tips and notifications aimed at improving sleep quality under heat stress.
2. Heat Protection at Work (3 features). This domain provides practical guidance to help workers protect themselves from heat exposure on the job. It includes recommendations for sun protection (sunscreen, sunglasses, hats), advice on selecting and using cooling devices tailored to personal needs, and best practices for managing work clothing, preparing spare garments, and reducing heat exposure in vehicles and air-conditioned environments.
3. Work Activity Planning (1 feature). This domain features a weather alert system integrated in the app, delivering push notifications with heat warnings and standardized advice (eg, “Tomorrow is expected to be hot; plan your breaks and bring sufficient water and fruit”). Unlike other services, these alerts will appear even if the app is closed or running in the background.
4. Refreshment and Leisure Time (2 features). These domains improve access to local resources for breaks at work and for leisure. An interactive, geo-located map highlights designated rest areas such as parks, cafes, shops, and libraries that offer sanitary facilities or access to potable water. Additional alerts provide information on nearby leisure facilities such as swimming pools and gyms, including opening hours and special offers through local partnerships.

### Data Collection Procedures

The impact of the REHEAT strategy is evaluated using data collected from June to September 2025 through a multi-source, multi-method data collection system integrated into the REHEAT app. The multi-source approach involves gathering data from various sources—including physiological parameters, self-reports, and environmental data—while the multi-method component includes passive data collection via portable sensors installed in Garmin smartwatches, online surveys and tests.

As shown in Figure 1, participants complete a pre-intervention on-boarding questionnaire (June 2025) that assesses their general quality of sleep, mental health, and physical health. This questionnaire also includes items on participants’ sociodemographic characteristics and prior work experience, with a particular focus on years of service, prior training, and personal heat mitigation coping strategies. The same instrument is administered again at the end of the trial (September 2025) as part of the off-boarding procedure to estimate the overall impact of the REHEAT strategy, calculated as the change in these outcomes over time between the treatment and control groups.

Data on participants’ strain accumulation and daily sleep are collected “passively” throughout the study via Garmin smartwatches. Cognitive performance, emotional distress, and night recovery are collected throughout the study via the REHEAT app, using short closed-ended questionnaires (“diaries”) and tests.

The off-boarding questionnaire issued toward the end of the RCT includes questions on health and well-being. It also encompasses a treatment-group-specific section assessing participants’ practical application of the app contents and their evaluation of its usefulness, with particular attention to their prior experience and competencies in managing heat. In addition, the off-boarding questionnaire includes targeted items to assess the risk of contamination among control group participants and to support potential adjustments for any under- or over-estimation of the effects of the REHEAT strategy.

Finally, the off-boarding questionnaire for the treatement group includes closed- and open-ended questions to assess participants’ experiences and satisfaction with the REHEAT strategy. The evaluation of the REHEAT strategy will be also supplemented by qualitative interviews with a small sub-sample (Autumn 2025).

### Participants Support and Monitoring

To promote adherence to the REHEAT intervention and ensure accurate data collection, a combination of participant support and monitoring strategies is implemented throughout the study.

A dedicated “Help” section is included in the REHEAT app, allowing users to access online guides and directly contact the research team via email to receive support throughout the study.

A standardized 3-level notification system is also implemented to keep in touch with and better assist users in completing their tasks. First, participants receive real-time notifications on their smartphones whenever new data collection tasks become available or when scheduled diaries or tests are overdue. Push notifications also alert participants if Garmin data cannot be retrieved, indicating potential issues with device use or synchronization with the Garmin Connect application. Second, participants receive automated weekly reports every Monday morning, providing individualized feedback on their task completion, including weekly response rates for diaries and the overall percentage of Garmin device wear, while also highlighting expected targets to motivate continued engagement. Third, an automated daily report is generated for the research team, providing summary information on participants who are experiencing delays with specific tasks. This enables the team to identify and contact these participants, understand the reasons for non-response, and provide the necessary support.

### Outcomes

**Figure 3.**
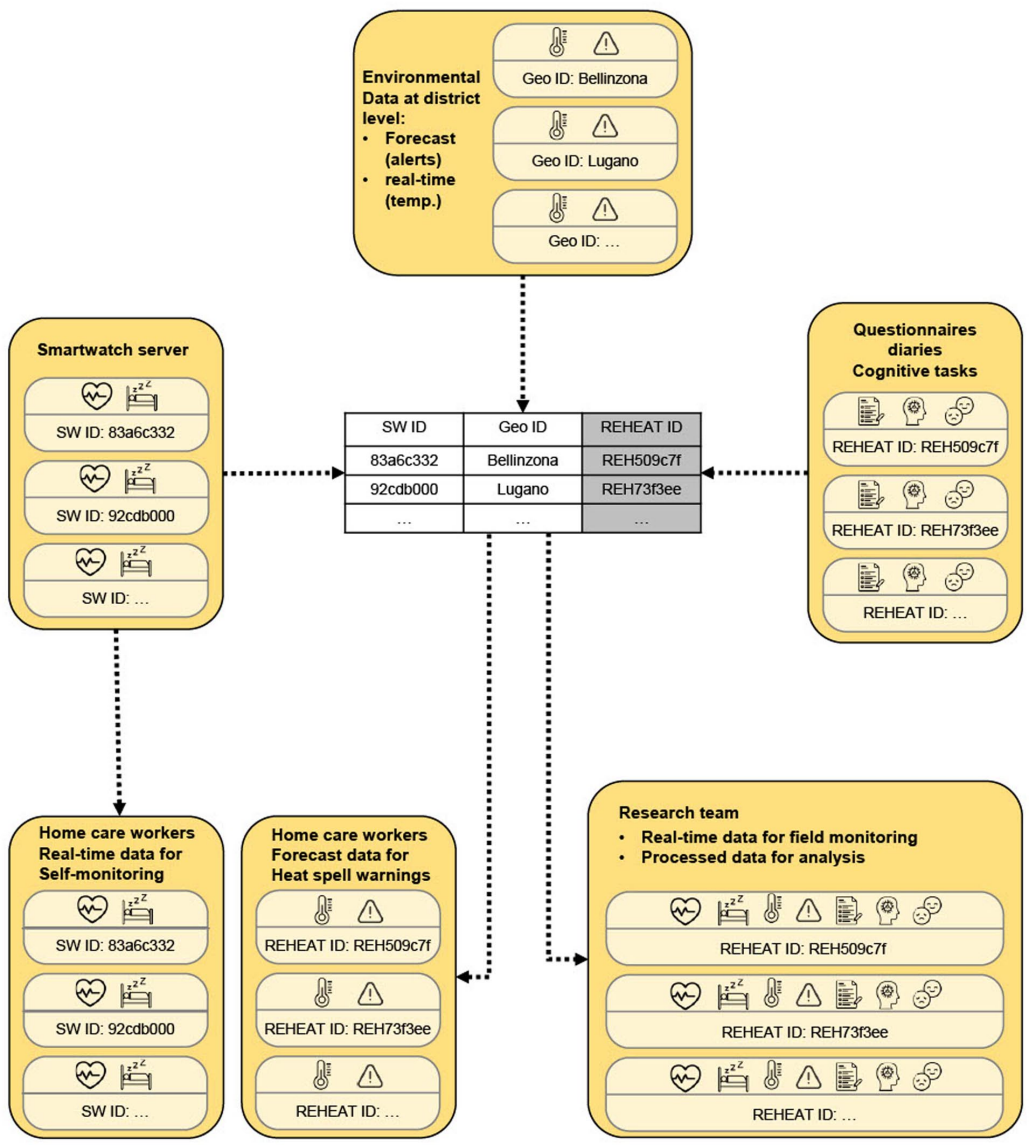
REHEAT data collection System and metrics.

The REHEAT app integrates a comprehensive data collection system to evaluate both the implementation and the impact of a heatwave adaptation strategy in the experimental groups. Data are collected through digital wearable devices with physiological sensors, app usage logs, online surveys and cognitive tests, and environmental monitoring (Figure 3). Smartphones and smartwatches enable the passive collection of behavioral and physiological data, which are combined with self-reported information to capture a rich, multidimensional picture of participants’ experiences.

A growing body of research highlights the advantages of integrating traditional survey methods with mobile and wearable technologies to gain more nuanced insights into individuals’ daily experiences and behaviors.^45^ These technologies support the continuous collection of high-resolution physiological and contextual data while minimizing the user burden, thus reducing recall bias and social desirability effects commonly associated with self-reports.^46^ The combination of “passive” sensing and traditional surveys allows for the joint analysis of objective indicators and subjective perceptions, including emotional states, routines, and situational appraisals.

The overall impact of the REHEAT strategy and the heterogeneity of its effects between groups is assessed through key primary outcomes, including strain accumulation, cognitive performance, emotional distress, sleep and night recovery, and mental and physical health. Secondary outcomes include objective and self-reported measures of intervention compliance, manipulation checks, and user experience and satisfaction. The complete list of study outcomes, along with detailed descriptions of their sources, data collection methods, and characteristics, is provided in Table 1.

**Table 1.** Summary of REHEAT Primary and Secondary Outcomes.

Outcome	Domain	Source	Time frame	Measurement description
*Primary outcomes*				
Strain accumulation	Physiological & psychological load	Garmin smartwatch; app-based questionnaires	Daily, with a focus on baseline vs heatwaves occurrence	Objective physiological heart rate metrics (HRV); self-reported stress (VAS)^47^ and daily activities
Cognitive performance	Cognitive function	App-based cognitive tests	Daily, with a focus on baseline vs heatwaves occurrence	Task-based cognitive performance via the short tests serial-seven^48^ and delayed recall^49^
Emotional distress	Psychological well-being	App-based questionnaires	Daily, with a focus on baseline vs heatwaves occurrence	Positive and Negative Affect (I-PANAS-SF)^50^
Sleep quality & quantity	Sleep and recovery	Garmin smartwatch; app-based questionnaires; pre- and post-intervention questionnaires	Daily, with a focus on baseline vs heatwaves occurrence; Middle term, pre- and post-intervention	Objective sleep duration and circadian rhythm; self-reported sleep quality and duration (B-PSQI, Karolinska sleep diary)^51,52^
Nighttime recovery	Physiological recovery	Garmin smartwatch; app-based questionnaires	Daily, with a focus on baseline vs heatwaves occurrence	Objective physiological heart rate metrics; self-reported recovery (Karolinska sleep diary)^51^
Mental & Physical Health	General health	App-based questionnaires; pre- and post intervention questionnaires	Daily, with a focus on baseline vs heatwaves occurrence; middle term, pre- and post-intervention	Self-reported injuries, general health, mental health (MHI-5),^53,54^ burnout (SMBM)^55^
*Secondary outcomes*				
Intervention Compliance	Engagement	App usage logs	Continuously during intervention	App interaction frequency and feature use tracked via in-app activity logs
Manipulation check	Health behavior	Garmin smartwatch; app-based questionnaires	Daily, with a focus on baseline vs heatwaves occurrence	Self-reported and objective indicators of coping behaviors for hydration, nutrition, peak-hour strenuous activity, etc.
User satisfaction & experience	User Experience	post-intervention questionnaire; interviews with subsample	Post-intervention	user experience questionnaire and qualitative interviews

### Sample Size Calculation

We estimate the minimum sample size for a 2-arm, parallel-group superiority RCT with a continuous outcome and 1:1 allocation using the standard formula:

$$n=\frac{2\sigma^{2}{\left(Z_{\alpha /2}+Z_{\beta }\right)}^{2}}{\Delta^{2}}$$

where *n* is the required sample size per group, *σ* is the standard deviation of the outcome, ∆ is the expected difference (minimum clinically important difference) between groups, *Z_α/_*_2_ is the critical value for a 2-sided significance level (*α* = .05), and *Z__β__* is the critical value corresponding to 80% power.

To inform the parameters *σ* and ∆, we use empirical data from a previous observational study conducted by the REHEAT research team on a sample of 294 office workers in Canton Ticino (Southern Switzerland), monitored for 30 working days between February and April 2024.^45^ In that study, daily recovery was defined as the normalized difference between so-called “body battery levels” (a measure assessed by means of smartwatches) in the evening and at wake-up. A fixed-effects panel regression model is estimated, with recovery in body battery levels as the dependent variable and average daily temperature (from the Lugano MeteoSwiss station) as the main predictor, controlling for perceived health, weekday, and week number.

Due to the absence of days with average temperatures above 18^◦^C in that dataset, we extrapolate the expected effect of hot days by assuming a linear relationship between temperature and recovery. We use predicted average recovery values for moderate days (14°C-18°C, typical of June in Canton Ticino) as the baseline and compare them to predicted values at 25°C—the MeteoSwiss threshold for heat episodes.^56^ The resulting difference in recovery is used as the expected intervention effect (∆), under the assumption that the REHEAT strategy could fully mitigate the adverse impact of heat.

Although optimistic, this assumption is conservative in terms of sample size estimation: the effect size is derived from a lower-risk population (office workers) and under the assumption of linearity, whereas prior literature suggests that the impact of heat on health is often nonlinear and intensifies at higher temperatures.

While the sample size is calculated based on a simple 2-sample comparison, the planned analyses will employ mixed-effects models, which account for repeated measures and hierarchical data structures and are therefore more complex than a simple *t*-test. To ensure adequate power and to compensate for potential design inefficiencies associated with these models, as well as expected dropout, we add a safety margin by increasing the total number of participants by about 10%.

Based on that analysis, we determine that a total sample ranging from approximately 330 to 370 participants would provide sufficient power to detect the estimated effect, with the upper bound reflecting an additional safety margin to account for potential dropout and model complexity.

### Statistical Analysis Plan

The evaluation of the REHEAT heat adaptation strategy consider the following 4 areas: implementation and compliance, impact, heterogeneity, and satisfaction.

- Implementation and compliance: descriptive statistics and inferential comparisons assess baseline equivalence between treatment and control group and participant compliance across relevant sociodemographic characteristics, work experience, health condition, and selected outcomes. App usage data is analyzed using frequency distributions and stratified analyses by age, sex, and location to ascertain user engagement with the app.
- Impact Analysis: The impact of the REHEAT strategy is first evaluated using an Intention-To-Treat (ITT) approach, which yields the most reliable estimates for real-world interventions by preserving the benefits of randomization. In parallel, Local Average Treatment Effects (LATE) are estimated to assess the intervention’s effects at varying levels of participants’ potential heat exposure and compliance. ITT analyses are conducted at the individual level. The effects of REHEAT on the primary outcomes—measured at on-boarding and off-boarding—as well as on those monitored throughout the summer, are estimated using random-intercept mixed-effects models. By accounting for within-subject correlations over time, this approach supports a robust analysis of longitudinal data. LATE estimates on primary outcomes are derived using instrumental variable generalized linear mixed models (IV-GLMM), with MeteoSwiss weather data and REHEAT app usage serving as instruments. Specifically, passively recorded data on the frequency of use of the app’s features over time are used as a proxy for participants’ compliance with the intervention. These models control for randomization strata and blocks, as well as a set of time-invariant and time-varying co-variates deemed theoretically or empirically relevant. Because heat effects vary across time, cumulative exposure, and acclimatization,^57,58^ temporal factors are explicitly controlled in the analyses. Exposure to heat is modeled using distributed lag models to capture both immediate and delayed effects of temperature and humidex across pre-specified lag periods. Mixed-effects models include calendar-week splines, random slopes for time, and adjustments for survey date and recent mean temperature to account for differences in measurement timing and seasonal acclimatization.^59^ Potential time-invariant characteristics of the respondents (eg, years of experience in home care, chronic conditions), and time-varying confounding due to intermediate variables (eg, workload changes, night shifts) are also addressed in sensitivity analyses.

- Effect Heterogeneity: interaction terms are included in multilevel models to explore differences in outcomes by sex, age, geographical region, and time-related factors. Separate models assess moderation effects by these variables in both ITT and LATE frameworks. Random intercept multilevel regression techniques are used to model hierarchical longitudinal data that violate the independence assumption (ie, repeatedly measured continuous outcomes). The heterogeneity of heat episode effects are estimated by multiple-interaction models in which interaction terms between the heat episode, the participant’s area of work, age, and sex are estimated. To capture temporal heterogeneity, additional interaction terms assess whether the REHEAT strategy differentially mitigates heat effects over the course of the summer. Treatment-by-time interactions, such as calendar week, cumulative heat exposure, or time since onboarding, are used to examine whether intervention effects vary due to seasonal acclimatization or repeated exposure.^59^ Flexible calendar-week splines and random slopes for time allow for individual adaptation trajectories and evolving heat vulnerability. These analyses determine whether the REHEAT strategy effectively addresses the diverse and changing needs of home care workers operating in different areas of Switzerland, who are exposed to different climatic, organizational and contextual conditions, while also estimating average conditional treatment effects across demographic and temporal dimensions.

- Satisfaction: Participants’ satisfaction is evaluated using a mixed-methods approach, combining quantitative survey data with qualitative insights. Qualitative data are collected through interviews with a small randomly selected sample of home care workers, drawn from the pool of study participants. The sampling ensures representation across key variables relevant to the study, such as geographical area, sex, and age. Selected individuals are contacted at the end of the study and invited to voluntarily share their experiences in either an online or face-to-face interview. Collected data are analyzed via thematic analysis, employing inductive coding strategies and inter-rater reliability checks. Quantitative data are obtained through a user experience questionnaire administered to all participants during the off-boarding phase, at the end of the RCT. The questionnaire aims to assess participants’ overall satisfaction and experience with the REHEAT study and, for those in the treatment group only, the perceived usefulness of the suggested REHEAT strategy.
- Contamination: Additionally, specific items are included in the offboarding questionnaire to gather information on any potential contamination that may occurr during the treatment period. Assessing contamination is essential in studies such as REHEAT that are conducted within organizational settings, where interactions among participants may lead to unintended exposure of control group members to elements of the intervention. Systematically examining potential contamination allows for a more accurate interpretation of the findings and strengthens the credibility of causal inferences.
- Handling of missing data: Missing data are addressed through multiple imputation using chained equations (MICE).^60^ For time-series of physiological data, additional methods such as temporal smoothing, regression-based imputation, and complete-case sensitivity analyses are used.

Quantitative analyses are carried out using STATA 19,^61^ R,^62,63^ and Python.^64^ Qualitative data are analyzed using dedicated software for qualitative research, specifically MAXQDA or NVivo.

### Data Management and Privacy Protection

The REHEAT study prioritizes rigorous data management and the highest standards of data privacy protection. Data collection involves both active input (eg, surveys, app diaries) and passive monitoring (eg, sensor data from the smartwatches). All personal data collected during the REHEAT study are pseudonymized at source and linked via unique participant identifiers (REHEAT IDs), which are securely stored and only accessible to authorized research staff.

Pseudonymized data from online questionnaires and in-app logs is directly stored on the secure servers at SUPSI. The decoding table linking personal information to internal REHEAT IDs is stored separately in a password-protected file on a secure cloud storage service designed specifically for higher education institutions in Switzerland. It is retained only until the end of the study and solely for the purpose of participant support.

The REHEAT app interfaces with the Garmin Health API to retrieve data collected and stored on Garmin servers, but only after participants have provided explicit consent via Garmin Connect. Once retrieved, the data are linked to a participant ID and thus pseudonymized. Garmin does not have access to any information collected specifically for the REHEAT project, except the data it gathers for the use of its wearable devices and API service, which is governed by the data protection agreement participants accept when using Garmin products. All data retrieved through the API are stored on secure, encrypted servers located at SUPSI in Switzerland. These servers comply with national and international data protection regulations, including the Swiss Federal Act on Data Protection (FADP) and the European Union General Data Protection Regulation (GDPR).

The data storage infrastructure includes:

- Separation of personal identifiers from research data;
- Encrypted communication protocols (HTTPS, SSL/TLS);
- Access controls with multi-factor authentication;
- Regular security audits and data access logs.

After completion of the REHEAT study, pseudonymized data and personal identification keys are stored for 20 years, in accordance with legal requirements. A processed version of the pseudonymized dataset is made publicly available on platforms such as Zenodo, adhering to FAIR data principles and ensuring no reasonable risk of re-identification for single REHEAT participants.

### Communication of Trial Results

The trial results are communicated to relevant stake-holders through multiple channels. A preliminary report summarizing data collection progress, including response rates, compliance, and user experience metrics, is disseminated late fall 2025 to study participants and participating organizations. This report also includes preliminary analyses of the full sample and subgroup analyses by organization. A comprehensive final impact evaluation report is prepared and distributed by late spring 2026, providing detailed findings on the intervention’s effectiveness. This report is supplemented by presentations aimed at organizational leaders and their employees to facilitate direct engagement and knowledge transfer. Findings are also presented and discussed with relevant institutional stake-holders and policymakers across Switzerland, to facilitate the operationalization and broader implementation of the strategy’s contents and support its scalability. In addition, a summary report is submitted to the ethics committee in accordance with regulatory requirements.

## Ethics and Monitoring

The REHEAT protocol is approved by the Ethics Committee of the Canton of Zurich (Kantonale Ethikkom-mission Zurich) and adheres to the latest version of the Declaration of Helsinki,^65^ ICH-GCP guidelines, and national legislation (HRA, ClinO).

Participant safety is monitored throughout the study. Although the intervention poses minimal risks to health and well-being, the study team monitors and reports all adverse events and serious adverse events (SAEs) in accordance with ClinO Articles 62 and 63. Annual safety and progress reports are submitted to the Ethics Committee.

Given the low-risk classification of the REHEAT trial, full external monitoring is not required. Instead, REHEAT adopts a hybrid model with:

- Three external monitoring visits (pre-, mid-, and post-intervention);
- Internal quality control based on the 4-eyes principle for data handling;
- Documentation audits and source data verification.

Additional internal checks include monitoring of randomization fidelity, participant consent compliance, and case report form accuracy. All monitoring activities are documented in the Trial Master File (TMF).

## Results

At the time of submission, recruitment had been successfully completed, with a total of 350 participants enrolled as planned. This number falls within the target range defined to ensure adequate statistical power and account for the applied safety margin. The intervention and data collection phases are ongoing, involving the continuous acquisition of physiological, environmental, and self-reported data through the REHEAT app and wearable sensors. Data quality checks and system monitoring procedures are currently in place to ensure completeness and reliability. No outcome data have yet been accessed or analyzed at this stage, and qualitative interviews with a sub-sample of participants are scheduled for Autumn 2025.

## Discussion

The REHEAT study addresses a critical and underexplored issue: the occupational health impacts of climate-induced heatwaves on home care workers. As climate extremes become more frequent, the health and safety of outdoor and mobile workers, particularly those providing essential services, will increasingly depend on effective heat adaptation strategies.^15,30^

This study has several notable strengths. First, it employs a multisite randomized controlled design with longitudinal and multi-modal data collection systems, offering strong internal validity. Second, the REHEAT strategy is co-designed with home care workers, their employers, and further experts, tailored to the unique context of Swiss home care sector, providing external validity. This enhances the feasibility for a potential permanent implementation of the REHEAT strategy and an up-scaling to other organizations or sectors.

Third, the integration of different data sources (eg, high-resolution wearable sensor data, self-reported information and environmental data from MeteoSwiss) enables a nuanced evaluation of physiological and psychological responses to heat exposure. This allows for real-time and temporally aligned assessments of both exposure and impact of the intervention.

If the REHEAT strategy proves to be effective, it can serve as a scalable model for outdoor or mobile workforces in other sectors, even beyond the health system. Scalability is further supported by the modular and digital nature of the REHEAT app, which can be adapted to other languages, regional climates, and occupational settings. Features such as automated alerts, context-specific guidance, and geo-located resources are transferrable to different user populations. Furthermore, the participatory design model used in REHEAT can serve as a template for user-centered development of digital health tools across domains.

Despite the strengths of its study design and intervention components, the REHEAT protocol may face some limitations. Contamination between treatment and control groups may occur due to shared workplace settings. Although partially mitigated through the app design and participant instructions, this could lead to underestimation of treatment effects. Future research could address this by scaling up the study and implementing a clustered design, randomizing entire organizations as treated or control units to reduce cross-group contamination.

The estimation of potential rather than actual heat exposure may also introduce non-differential measurement error, attenuating observed effects. In this study, daily potential exposure was estimated using meteorological data from each participant’s local administrative unit, within-subject variations were analyzed over time, and time- and weather-related covariates controlled for. To our knowledge, no wearable devices currently exist that can reliably measure environmental temperature and humidity over extended periods for homecare workers during and outside work. Future studies will require such tools to capture real heat exposure, improving exposure assessment and strengthening causal inference without relying on self-reported data.

Finally, external validity may be limited to the Swiss context, as the intervention’s effectiveness could be influenced by substantial heterogeneity across geographical areas. Differences in local infrastructure, organizational practices, and cultural contexts, as well as variations in prior heat exposure and climate habituation, may all shape both risk perception and responses to the intervention. Recognizing these sources of heterogeneity is important for interpreting the results and for planning future studies in different settings or populations to generalize them.

## Conclusion

The REHEAT study provides timely and urgently needed evidence on the effectiveness of targeted, co-designed digital strategies to protect home care workers from heat-related health risks caused by climate change. By integrating wearable sensors, self-reports, and environmental data, the trial assesses both physiological and psychological outcomes. The study demonstrates how digital technologies can support behavioral and organizational responses to environmental stressors, offering a model for adaptive, data-informed, and user-centered interventions. Findings will inform occupational health guidelines and policies, contribute to the development of scalable strategies for other mobile work-forces, and support broader public health initiatives aimed at human adaptation to climate extremes.^66^

## Supplemental Material

sj-docx-1-inq-10.1177_00469580251405766 – Supplemental material for An App-Based Intervention to Protect Home Care Workers From Heat-Related Health Risks: Protocol for a Randomized Controlled Trial Supplemental material, sj-docx-1-inq-10.1177_00469580251405766 for An App-Based Intervention to Protect Home Care Workers From Heat-Related Health Risks: Protocol for a Randomized Controlled Trial by Tiziano Gerosa, Remo Fortunato, Francesca Cellina, Martina Ragettli, Davide Marzorati, Andrea Baldassari, Sara Levati, Azra Karabegovic and Isabel Baumann in INQUIRY: The Journal of Health Care Organization, Provision, and Financing
